# Wild-Type Transthyretin Cardiac Amyloidosis Presenting As Progressive Heart Failure and Conduction Disease Despite Guideline-Directed Medical Therapy

**DOI:** 10.7759/cureus.109667

**Published:** 2026-05-26

**Authors:** Srivane Richard, Dea Thomas, Asher Gorantla, Sameeksha Devkota, Suzette Graham-Hill

**Affiliations:** 1 Department of Internal Medicine, State University of New York Downstate Health Sciences University, Brooklyn, USA; 2 Department of Cardiology, State University of New York Downstate Health Sciences University, Brooklyn, USA

**Keywords:** cardiac amyloidosis, cardiac mri, conduction disease, heart failure, infiltrative cardiomyopathy, tafamidis, wild-type transthyretin amyloidosis

## Abstract

Wild-type transthyretin cardiac amyloidosis (ATTRwt) is an underrecognized cause of heart failure that often mimics hypertensive or nonischemic cardiomyopathy. A 70-year-old woman with hypertension, diabetes, and chronic kidney disease presented with dyspnea, reduced left ventricular ejection fraction, and a 2:1 block requiring dual-chamber pacemaker implantation. Despite guideline-directed medical therapy, her heart failure worsened with further decline in systolic function. Ischemic etiology was excluded. Cardiac magnetic resonance demonstrated diffuse subendocardial late gadolinium enhancement consistent with amyloidosis. Negative monoclonal protein studies and genetic testing confirmed ATTRwt. Her device was subsequently upgraded to a cardiac resynchronization therapy-defibrillator due to persistent ventricular dysfunction and high pacing burden. Tafamidis was then initiated. This case highlights the limitations of conventional heart failure therapy in amyloid cardiomyopathy and the importance of early imaging-based diagnosis for timely disease-directed treatment.

## Introduction

Cardiac amyloidosis is an infiltrative cardiomyopathy caused by extracellular deposition of misfolded protein fibrils, leading to progressive ventricular dysfunction, conduction abnormalities, and heart failure. Wild-type transthyretin cardiac amyloidosis (ATTRwt) is increasingly recognized in older adults and is frequently misdiagnosed as hypertensive or nonischemic cardiomyopathy because of overlapping clinical and echocardiographic features [1]. Conduction disease, atrial arrhythmias, and unexplained ventricular thickening may precede overt heart failure and should raise suspicion for infiltrative disease [2]. Early recognition of ATTRwt has become increasingly important because disease-specific therapy with Tafamidis can slow disease progression and improve clinical outcomes, whereas conventional heart failure therapy often provides limited benefit [3]. Here, a case is reported of ATTRwt presenting with atrioventricular conduction disease and progressive systolic heart failure despite pacemaker implantation and guideline-directed medical therapy.

## Case presentation

A 70-year-old woman with longstanding hypertension, type 2 diabetes mellitus, and stage 3 chronic kidney disease presented with several months of exertional dyspnea, reduced exercise tolerance, fatigue, and intermittent lightheadedness. She denied chest pain, syncope, orthopnea, or lower extremity edema at initial presentation.

Physical examination

Vital signs demonstrated a blood pressure of 124/76 mmHg with a heart rate of 52 beats/minute and no hypoxia. Physical examination revealed regular rhythm without murmurs, mild bibasilar crackles, and no significant peripheral congestion.

Investigations

Electrocardiography demonstrated a 2:1 block with blocked premature atrial contractions (Figure 1).

**Figure 1:**
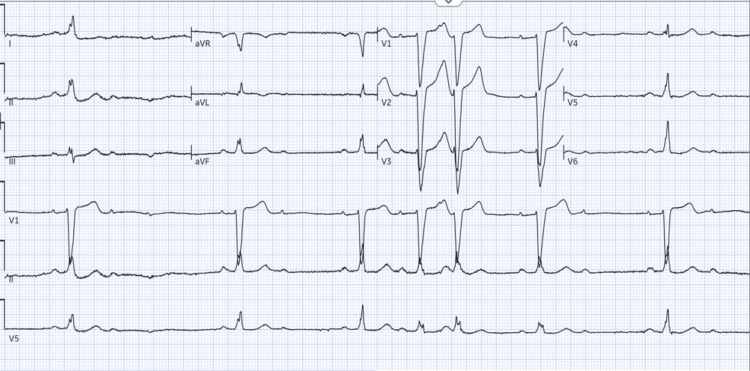
Electrocardiogram showing 2:1 block with blocked premature atrial contractions aVR: augmented vector right; aVL: augmented vector left; aVF: augmented vector foot

Transthoracic echocardiography showed concentric left ventricular (LV) wall thickening, biatrial enlargement, global hypokinesis, and reduced LV ejection fraction estimated at 30%-35%. Right ventricular (RV) systolic function was mildly reduced, and diastolic filling parameters suggested elevated filling pressures. The wall thickening was initially attributed to chronic hypertensive remodeling.

Initial management

A dual-chamber permanent pacemaker was implanted because of symptomatic high-grade conduction disease (Figure 2). Cardiac resynchronization therapy defibrillator therapy was deferred as LV dysfunction may have been potentially reversible.

**Figure 2:**
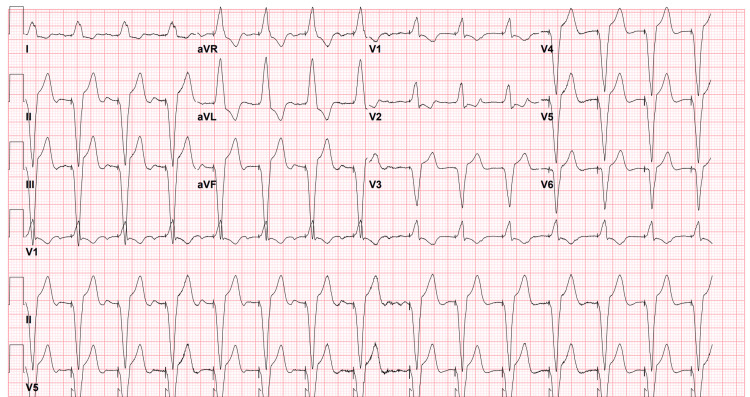
Electrocardiogram showing a ventricular paced rhythm following pacemaker implantation aVR: augmented vector right; aVL: augmented vector left; aVF: augmented vector foot

Following device placement, she was started on conventional heart failure therapy, including a low-dose beta-blocker, renin-angiotensin system blockade, mineralocorticoid receptor antagonist, and loop diuretic as tolerated, with dose adjustment limited by renal dysfunction and borderline blood pressure.

Postdischarge course, rehospitalization, and subsequent diagnostic and therapeutic interventions

Over the following months, she developed worsening exertional intolerance with recurrent dyspnea and lower extremity edema, prompting rehospitalization for acute decompensated heart failure. Laboratory studies demonstrated elevated natriuretic peptide levels to 5,500 pg/mL (reference level <100 pg/mL) and stable chronic kidney dysfunction without acute ischemic biomarker elevation. Repeat transthoracic echocardiography revealed further decline in LV ejection fraction to 25%, persistent concentric thickening, restrictive filling pattern, and worsening atrial enlargement (Figure 3).

**Figure 3:**
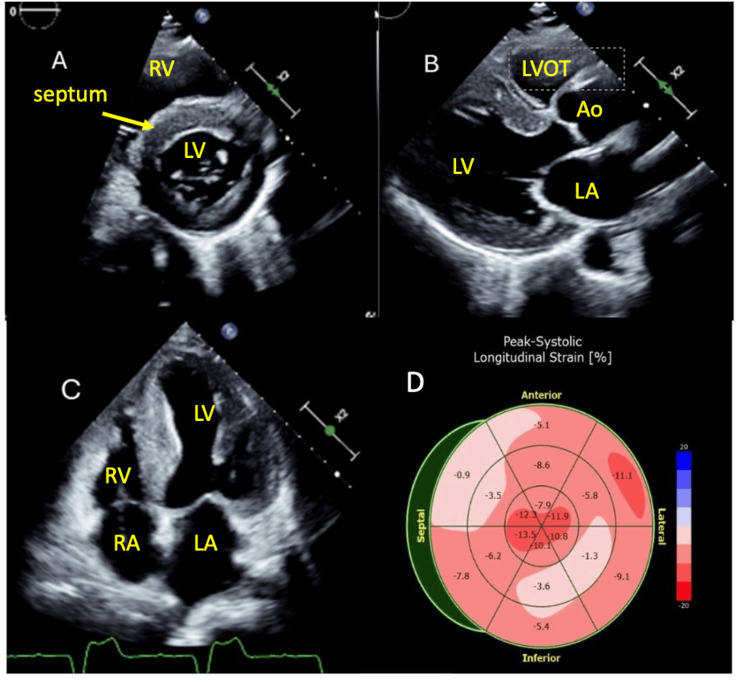
Transthoracic echocardiography showing (A) parasternal short axis, (B) parasternal long axis, and (C) apical four-chamber views all showing increased left ventricular thickness. (D) Global longitudinal strain with apical sparing Ao: aorta; LV: left ventricle; RV: right ventricle; RA: right atrium; LVOT: left ventricular outflow track; LA: left atrium

Given the progressive decline despite medical therapy, ischemic evaluation was pursued. A nuclear myocardial perfusion study was performed, which showed a small area of scar in the apex, but overall, there was no evidence of ischemia.

The combination of progressive heart failure, conduction disease, ventricular thickening disproportionate to the patient’s blood pressure history, and absence of ischemic substrate raised concern for infiltrative cardiomyopathy. The patient then underwent a cardiac pyrophosphate (PYP) scan, which demonstrated diffuse circumferential subendocardial late gadolinium enhancement involving both ventricles, markedly abnormal myocardial nulling, and diffuse extracellular expansion, findings strongly suggestive of cardiac amyloid infiltration (Figure 4).

**Figure 4:**
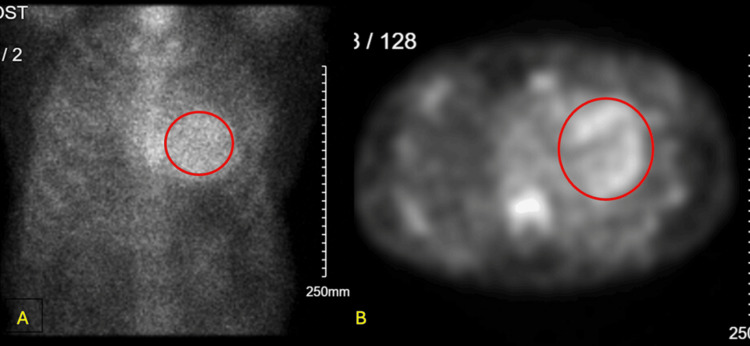
Pyrophosphate imaging. Technetium-99m pyrophosphate acquired three hours after injection showing (A) plantar anterior visual and (B) single-photon emission computerized tomography visual demonstrating diffuse circumferential subendocardial late gadolinium enhancement involving both ventricles (outlined by red circle) and correlating to Grade 3 visual score

Serum-free light chains, serum immunofixation electrophoresis, and urine immunofixation were negative for monoclonal protein, reducing the likelihood of light-chain amyloidosis. Subsequent transthyretin genetic testing showed no pathogenic mutation, confirming ATTRwt.

Her device was interrogated, which demonstrated a high burden of RV pacing with progressive LV dysfunction consistent with pacing-induced desynchrony. Given these findings, amyloid cardiomyopathy is particularly vulnerable to the adverse hemodynamic effects of chronic RV pacing; her pacemaker was upgraded to cardiac resynchronization therapy with defibrillator support (Figure 5).

**Figure 5:**
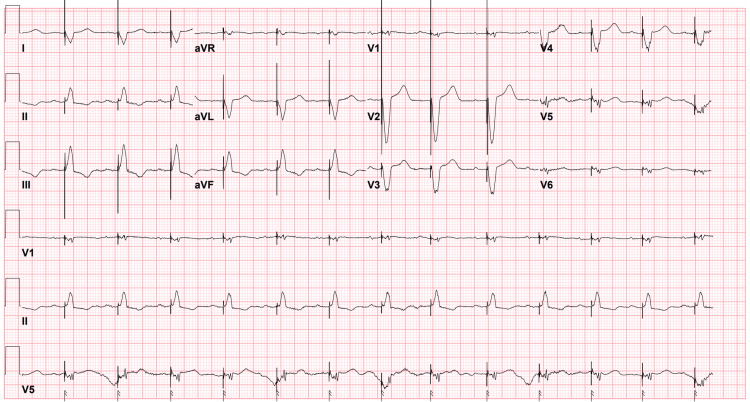
Electrocardiogram showing both A (atrial) and V (ventricular) paced rhythm following device upgrade to cardiac resynchronization therapy defibrillator aVR: augmented vector right; aVL: augmented vector left; aVF: augmented vector foot

Following diagnostic confirmation, treatment with tafamidis was initiated for disease modification. During outpatient follow-up, device interrogation identified short episodes of asymptomatic atrial fibrillation. Given the elevated thromboembolic risk associated with amyloid cardiomyopathy, anticoagulation was initiated despite a brief arrhythmia burden. Functional symptoms stabilized after device upgrade and targeted therapy, with reduced heart failure admissions during subsequent follow-up.

She was seen in the outpatient heart failure clinic for three months with improvement in heart failure symptoms and a repeat transthoracic echocardiogram showing an LV ejection fraction of 31%. The timeline from the patient's clinical presentation to diagnostic workup and therapeutic interventions has been summarized in Table 1.

**Table 1:** Timeline of the clinical presentation, diagnostic workup, and therapeutic interventions CRT-D: cardiac resynchronization therapy defibrillator

Time point	Clinical course
Initial presentation	Progressive dyspnea, fatigue, and symptomatic Mobitz II atrioventricular block
Initial echocardiography	Left ventricular ejection fraction 30%-35% with concentric left ventricular thickening
Early intervention	Dual-chamber permanent pacemaker implantation and initiation of guideline-directed medical therapy
Follow-up hospitalization	Recurrent decompensated heart failure with worsening exertional intolerance
Repeat echocardiography	Decline in left ventricular ejection fraction to 25%
Ischemic evaluation	Nuclear myocardial perfusion imaging negative for reversible ischemia
Advanced imaging	Cardiac magnetic resonance demonstrating diffuse subendocardial late gadolinium enhancement consistent with infiltrative cardiomyopathy
Laboratory evaluation	Negative serum and urine monoclonal protein studies excluding light-chain amyloidosis
Genetic evaluation	Negative transthyretin mutation confirming wild-type transthyretin amyloidosis
Definitive management	Upgrade to CRT-D and initiation of tafamidis

## Discussion

ATTRwt is an age-related infiltrative cardiomyopathy. Unlike hereditary transthyretin amyloidosis, ATTRwt occurs in the absence of a pathogenic transthyretin mutation and predominantly affects older adults, with prevalence increasing significantly after 65 years of age. Contemporary screening studies suggest that ATTRwt may account for a meaningful proportion of unexplained heart failure in elderly patients, particularly those with increased LV wall thickness, conduction abnormalities, or low-flow aortic stenosis, indicating that the disease is substantially underrecognized in routine practice [1,2].

A major challenge in ATTRwt remains delayed diagnosis because its clinical presentation often overlaps with hypertensive heart disease, hypertrophic remodeling, and nonischemic cardiomyopathy. Patients frequently present with nonspecific symptoms such as exertional dyspnea, fatigue, reduced exercise tolerance, or conduction disease before overt restrictive physiology develops. In this case, a 2:1 block with blocked premature atrial contractions requiring permanent pacing due to symptomatic presentation preceded definitive recognition of infiltrative cardiomyopathy, which is a well-described but often overlooked manifestation of amyloid infiltration of the conduction system. Although echocardiography may show concentric ventricular thickening, biatrial enlargement, diastolic dysfunction, and reduced longitudinal strain with apical sparing, these findings are not specific and may initially be attributed to chronic hypertension or other myocardial disease [3]. Electrocardiographic low voltage, classically associated with amyloidosis, is present in only a minority of patients and therefore cannot reliably exclude the diagnosis [1,4].

The current American College of Cardiology expert consensus recommends a stepwise diagnostic approach beginning with exclusion of light-chain amyloidosis using serum-free light chain assay together with serum and urine immunofixation electrophoresis, since missing light-chain amyloidosis carries major therapeutic consequences [3]. Serum or urine protein electrophoresis alone is insufficient due to its lower diagnostic sensitivity. Once monoclonal protein studies are negative, transthyretin amyloidosis should be evaluated with bone scintigraphy using technetium-labeled PYP imaging or advanced tissue characterization with cardiac magnetic resonance imaging [5]. Cardiac magnetic resonance is particularly valuable when diffuse subendocardial late gadolinium enhancement, abnormal myocardial nulling, and extracellular volume expansion are present, all of which strongly support infiltrative amyloid cardiomyopathy, as demonstrated in this patient [3,6]. Genetic testing is then required to distinguish wild type from hereditary transthyretin disease after ATTR is identified [3].

Management of ATTRwt differs substantially from conventional heart failure treatment because standard neurohormonal therapies are often poorly tolerated and have limited disease-modifying effects. Beta-blockers may worsen low-output symptoms. Angiotensin-converting enzyme inhibitors and angiotensin receptor blockers frequently cause hypotension because of fixed stroke volume and autonomic dysfunction, and aggressive vasodilation may further compromise cardiac output. Diuretics, therefore, remain the cornerstone of symptomatic volume management, while mineralocorticoid receptor antagonists may be used cautiously when renal function allows [3]. In this patient, progressive decline despite guideline-directed heart failure therapy reflected the inability of conventional treatment to halt amyloid progression.

Disease-directed therapy now centers on tafamidis, a transthyretin stabilizer that prevents tetramer dissociation and subsequent fibril formation. Tafamidis is currently the only FDA-approved therapy for transthyretin cardiac amyloidosis and is recommended for symptomatic ATTR cardiomyopathy in New York Heart Association class I through III because it reduces all-cause mortality and cardiovascular hospitalization while slowing functional decline [7]. Importantly, tafamidis delays progression rather than reverses established myocardial infiltration, making early diagnosis essential for maximal benefit [8,9]. Diflunisal may be considered as an alternative transthyretin stabilizer when tafamidis is inaccessible, although it is often avoided in patients with chronic kidney disease or decompensated heart failure because of renal and gastrointestinal toxicity [3]. Emerging therapies, including gene silencers such as patisiran and vutrisiran, continue to show promise, but current guideline-based use remains more established in hereditary neuropathic disease than isolated wild-type cardiomyopathy [4].

Arrhythmia and device management also require special consideration in ATTRwt. Permanent pacing is frequently necessary because conduction disease is common, but chronic RV pacing may worsen ventricular desynchrony and contribute to progressive systolic dysfunction. In this patient, high pacing burden likely accelerated ventricular deterioration. Amyloid cardiomyopathy is particularly vulnerable to the adverse hemodynamic effects of chronic RV pacing, as this creates ventricular dyssynchrony similar to left bundle branch block physiology. In amyloid hearts, even modest dyssynchrony may cause worsening LV systolic function, decline in cardiac output, worsening diastolic dysfunction, and progressive heart failure symptoms. Due to these concerns, her device was upgraded to a cardiac resynchronization therapy-defibrillator. Defibrillator implantation in amyloid cardiomyopathy remains individualized because sudden death often occurs from electromechanical dissociation rather than ventricular tachyarrhythmia, and survival benefit has not been consistently demonstrated beyond standard heart failure indications [3].

Atrial fibrillation is highly prevalent in ATTRwt and carries an elevated thromboembolic risk independent of conventional risk scores. Current consensus, therefore, recommends anticoagulation for any documented atrial fibrillation regardless of CHA2DS2-VASc (congestive heart failure, hypertension, age ≥75 years, diabetes mellitus, stroke, vascular disease, age 65-74 years, and sex category) score, even when episodes are brief or asymptomatic, as intracardiac thrombus formation occurs disproportionately in amyloid cardiomyopathy [3,10]. This guided anticoagulation after device-detected atrial fibrillation in our patient.

Overall, this case emphasizes that ATTRwt should be suspected early in older patients with unexplained conduction disease, ventricular thickening, and progressive heart failure despite standard therapy, because earlier recognition directly determines access to disease-modifying treatment and improved long-term outcomes.

## Conclusions

ATTRwt should be strongly considered in older adults with unexplained heart failure, conduction disease, ventricular thickening, or progressive decline despite conventional therapy. This case demonstrates that standard heart failure management and pacemaker implantation may not halt disease progression when amyloid cardiomyopathy remains unrecognized. Early multimodality imaging, exclusion of light-chain disease, and prompt initiation of transthyretin-targeted therapy are essential because earlier diagnosis directly influences prognosis and hospitalization burden.
